# Особенности субпопуляционного состава регуляторных Т-клеток крови и уровень экспрессии CD25 у пациентов с болезнью Грейвса в динамике после радионуклидного лечения

**DOI:** 10.14341/probl13223

**Published:** 2023-06-30

**Authors:** М. А. Дудина, А. А. Савченко, С. А. Догадин, А. Г. Борисов, В. Д. Беленюк

**Affiliations:** Красноярский государственный медицинский университет им. профессора В.Ф. Войно-Ясенецкого; Краевая клиническая больница; Красноярский государственный медицинский университет им. профессора В.Ф. Войно-Ясенецкого; Красноярский научный центр Сибирского отделения Российской академии наук; Красноярский государственный медицинский университет им. профессора В.Ф. Войно-Ясенецкого; Краевая клиническая больница; Красноярский государственный медицинский университет им. профессора В.Ф. Войно-Ясенецкого; Красноярский научный центр Сибирского отделения Российской академии наук; Красноярский научный центр Сибирского отделения Российской академии наук

**Keywords:** болезнь Грейвса, регуляторные Т-лимфоциты, уровень экспрессии CD25, радионуклидная терапия

## Abstract

**ОБОСНОВАНИЕ:**

ОБОСНОВАНИЕ. Пониженное число регуляторных Т-клеток (Treg) на разных стадиях формирования эффекторных субпопуляций и уровень экспрессии CD25 на их мембране при болезни Грейвса (БГ) могут определять длительную персистенцию аутоиммунного процесса и являться мишенью для иммунотропной терапии заболевания.

**ЦЕЛЬ:**

ЦЕЛЬ. Изучить особенности субпопуляционного состава и уровень экспрессии CD25 на мембране Treg в крови у пациентов с БГ в динамике после радионуклидного лечения для выделения отдельных субпопуляций Treg в качестве возможных мишеней для иммунотропной терапии заболевания.

**МАТЕРИАЛЫ И МЕТОДЫ:**

МАТЕРИАЛЫ И МЕТОДЫ. Проведено одноцентровое проспективное когортное открытое контролируемое исследование с участием пациентов с лабораторно подтвержденной БГ. Методом проточной цитометрии с применением моноклональных антител были исследованы особенности субпопуляционного состава Treg и уровень экспрессии MFI поверхностного рецептора СD25.

**РЕЗУЛЬТАТЫ:**

РЕЗУЛЬТАТЫ. В исследование включены 36 женщин с рецидивом БГ, средний возраст 46,34±14,32 года. У пациентов с БГ до и в течение всего периода после радионуклидного лечения установлен низкий относительно контрольного диапазона процентный уровень наивных (CD45R0-CD62L+) и терминально-дифференцированных (CD45R0-CD62L-) Treg, а через 3 и 6 мес выявлено значительное снижение клеток с данным фенотипом и относительно значений, выявляемых у больных в период до и через 1 мес после радиойодтерапии (p<0,001). Наиболее значимые изменения в уровнях экспрессии рецептора CD25 были обнаружены через 3 и 6 мес после радионуклидного лечения: на фоне компенсированного пострадиационного гипотиреоза в крови пациентов с БГ остаются пониженными уровни MFI CD25 на поверхности наивных и терминально-дифференцированных Treg.

**ЗАКЛЮЧЕНИЕ:**

ЗАКЛЮЧЕНИЕ. Установлено понижение уровня наивных Treg (по-видимому, за счет нарушения процессов дифференцировки в тимусе) и терминально-дифференцированных Treg (за счет процессов созревания и выживаемости), которые дополняются пониженным уровнем экспрессии рецептора CD25 на поверхности этих клеток и не зависят от компенсации гипертиреоза, титра антител к рецептору тиреотропного гормона, предшествующей консервативной терапии тиамазолом и радионуклидного лечения. Полученные новые данные вносят вклад в понимание роли наивных и терминально-дифференцированных cубпопуляций Treg в иммунопатогенезе БГ и помогают наметить дальнейшие пути разработки подходов для иммунотропной терапии.

## Abstract (en)

**BACKGROUND:**

BACKGROUND: The content of regulatory T cells (Treg) at different stages in formation of effector subpopulations and the level of CD25 expression on the membrane of their various fractions in Graves’ disease can determine the long-term autoimmune process persistence and be the target of immunotropic therapy of the disease.

**AIM:**

AIM: To study the features of regulatory T-blood cells subpopulation and the level of CD25 expression in patients with Graves’ disease in dynamics after radioactive iodine therapy (RIT) to identify the specific Treg subpopulations for potential immunotropic therapy targets of the disease.

**MATERIALS AND METHODS:**

MATERIALS AND METHODS: A single-center, prospective, cohort, open, controlled study was conducted with the participation of women with laboratory-confirmed Graves’ disease. The features of regulatory T-blood cells subpopulation and the level of expression (MFI) CD25 surface receptor were studied by flow cytometry using direct immunofluorescence using monoclonal antibodies.

**RESULTS:**

RESULTS: The study included 36 women with recurrent Graves’ disease, middle age 46.34±14.32 years. In patients with Graves’ disease before and during the entire period after RIT a low percentage of naive (CD45R0-CD62L+) and terminally differentiated (CD45R0-CD62L-) Treg was established relative to the control, and on 3 and 6 months after RIT a significant decrease of cells with this phenotype was revealed relative to the values detected in patients before and 1 month after RIT (p<0.001). Against the background of compensated hypothyroidism the most significant changes of expression CD25 receptor in patients with Graves’ disease were found on 3 and 6 months after RIT: reduced levels of MFI CD25 on surface of naive and terminally differentiated Treg.

**CONCLUSION:**

CONCLUSION: A decrease in the level of naive Treg was found (apparently due to a violation of differentiation processes in thymus) and terminally differentiated Tregs (due to maturation and survival processes), which are supplemented by a reduced expression of the CD25 receptor on the surface of these cells and do not depend on hyperthyroidism compensation, the titer of TSH receptor antibodies, previous conservative therapy with thiamazole and RIT. The obtained new data reveal the role of naive and terminally differentiated Treg subpopulations in immunopathogenesis and help to outline further ways to develop approaches for immunotropic therapy.

## ОБОСНОВАНИЕ

Болезнь Грейвса (БГ) — органоспецифическое аутоиммунное заболевание, возникающее вследствие нарушения иммунологической толерантности и образования аутоантител к рецептору тиреотропного гормона (рТТГ), клинически выражающееся поражением щитовидной железы (ЩЖ) с формированием синдрома тиреотоксикоза и экстратиреоидной патологией. Ежегодно регистрируется 20–50 новых случаев на 100 тыс. человек, при этом заболевание превалирует среди женщин [1][2]. По завершении консервативной терапии всего 30% пациентов достигают стойкой ремиссии, в то время как у большинства пациентов с БГ заболевание рецидивирует и клинически может проявляться как гипертиреозом, так и сменой медикаментозного гипо- и эутиреоза при консервативном лечении антитиреоидными препаратами [3][4].

Расширение знаний о роли регуляторных Т-лимфоцитов (Treg) и дефекте их супрессорной функции, а также фенотипическая изменчивость и высокая конститутивная экспрессия поверхностного маркера CD25 (рецептора для интерлейкина 2, IL-2R) имеют решающее значение в раскрытии механизмов персистенции аутоиммунного процесса при БГ [1][5]. Treg реализуют супрессию, используя многочисленные механизмы, причем их важной конститутивной фенотипической особенностью является высокий уровень экспрессии маркера CD25 [6].

К настоящему времени Treg рассматриваются как возможная терапевтическая мишень при БГ. В некоторых исследованиях в качестве мишени иммунотропной терапии заболевания обсуждается cубпопуляция адаптивных Treg, дефект которых при БГ заключается в отсутствии признаков пролиферации и продукции эффекторных цитокинов при их стимуляции [7][8]. Однако до сих пор отсутствует полное понимание механизмов снижения уровня и дефекта супрессорной функции Treg при БГ [9]. В результате неэффективности консервативной терапии возникает необходимость в разработке этиопатогенетических методов лечения БГ. С учетом возможного влияния консервативной терапии тиамазолом и радионуклидного лечения на иммунный статус пациентов с БГ, актуальным является изучение уровня Treg в динамике после проведенного лечения [10].

Содержание Treg на разных стадиях формирования эффекторных субпопуляций и уровень экспрессии CD25 на их мембране могут определять длительную персистенцию аутоиммунного процесса при БГ. В свою очередь, изучение cодержания отдельных субпопуляций Treg является многообещающим подходом для будущих методов лечения заболевания.

## ЦЕЛЬ ИССЛЕДОВАНИЯ

Изучить особенности субпопуляционного состава и уровень экспрессии CD25 на мембране Treg в крови у пациентов с БГ в динамике после радионуклидного лечения для выделения отдельных субпопуляций Treg в качестве возможных мишеней для иммунотропной терапии заболевания.

## МАТЕРИАЛЫ И МЕТОДЫ

## Место и время проведения исследования

Место проведения. Исследование проводилось на базе эндокринологического центра КГБУЗ «Краевая клиническая больница» (г. Красноярск).

Время исследования. Наблюдение пациентов осуществлялось с 6 cентября 2017 г. по 24 марта 2021 г.

## Изучаемые популяции

Изучались две популяции — пациенты с БГ и здоровые люди (контроль).

Популяция «пациенты с БГ»

Критерии включения: женский пол, возраст от 18 до 65 лет, лабораторно подтвержденная БГ.

Популяция «контроль»

Критерии включения: женский пол, возраст от 18 до 65 лет.

Критерии исключения в изучаемых популяциях: узловой/многоузловой токсический зоб, беременность, лактация, эндокринная офтальмопатия, наличие инфекционных и аллергических заболеваний, новообразования, системные заболевания соединительной ткани, другие заболевания органов эндокринной системы, острые респираторные и вирусные инфекции, сердечная недостаточность, введение профилактических прививок в течение 2 мес, предшествующих иммунологическому и гормональному анализу.

## Способ формирования выборки из изучаемой популяции

Формирование группы больных с рецидивом БГ проводилось сплошным способом подбора. Верификация диагноза, консервативное лечение и направление пациентов с БГ на радионуклидное лечение осуществлялись согласно национальным клиническим рекомендациям [11]. Рецидивом гипертиреоза считалось изменение показателей тиреоидного статуса, соответствующих критериям субклинического или манифестного гипертиреоза, у обследуемых пациентов с ранее достигнутым и подтвержденным лабораторно медикаментозным эутиреозом.

## Дизайн исследования

Проведено одноцентровое проспективное когортное открытое контролируемое исследование с участием пациентов с лабораторно подтвержденной БГ.

## Методы

Все обследуемые пациенты наблюдались в КГБУЗ «Краевая клиническая больница» с дебюта заболевания и получали медикаментозное лечение тиамазолом по стандартной схеме с постепенным переходом на поддерживающую дозу тиреостатика. При диагностике рецидива гипертиреоза до устранения симптомов тиреотоксикоза всем пациентам доза тиамазола была увеличена до лечебной. Поддерживающая доза тиамазола отменялась за 14 дней до приема раствора радиоактивного йода (131I). Клинико-гормональное и иммунологическое обследования проводились до приема терапевтической активности 131I, а также через 1, 3 и 6 мес после радионуклидного лечения. Обследование пациентов с БГ до радиойодтерапии (РЙТ) проводилось непосредственно в день проведения процедуры, соответственно через 2 нед после отмены тиамазола. Всем пациентам с диагностируемым пострадиационным гипотиреозом назначалась заместительная терапия левотироксином натрия.

Клинические методы обследования включали объективный осмотр, пальпацию ЩЖ, клинико-лабораторную оценку тиреоидного статуса и титра антител к рТТГ в сыворотке крови до и в динамике после радионуклидного лечения. Определение содержания тиреоидных гормонов осуществлялось методом хемилюминесцентного иммуноанализа на автоматическом анализаторе Architecti 1000sr (Abbott Diagnostics, США) в гормональной лаборатории эндокринологического центра Красноярской краевой клинической больницы, референсные показатели: для ТТГ (0,4–4,0 мЕд/л), свободного тироксина (свТ4) (9,01–19,05 пмоль/л), свободного трийодтиронина (свТ3) (2,14–6,42 пмоль/л). Уровень аутоантител к рТТГ оценивался иммуноферментным методом при помощи набора Medizym T.R.A. (MedipanGmbH, Германия). Исследование объема и определение структуры ЩЖ у пациентов с БГ и лиц контрольной группы проводилось ультразвуковым методом на аппарате Philips iU22 xMatrix (США) с линейным датчиком 7,5 МГц.

Радионуклидное лечение проводилось на базе отделения радионуклидной терапии ФМБА России г. Красноярска. Для всех пациентов радионуклидное лечение проводилось впервые в качестве терапии второй линии. На основании комплексного предтерапевтического обследования всем пациентам назначалась удельная активность 131I перорально в виде изотонического водного раствора натрия йодида. Исследование фенотипа Т-лимфоцитов проводили методом проточной цитометрии цельной периферической крови с применением моноклональных антител (Beckman Coulter, USA), меченных FITC (fluorescein isothiocyanate), PE (phycoerythrin), ECD (phycoerythrin-TexasRed-X), PC5 (phycoerythrin-cyanin 5), PC7 (phycoerythrin-cyanin 7), AA700 (alexafluor 700) и AA750 (alexafluor 750) в следующих панелях: CD8-FITC/CD127-PE/CD25-PC5/CD4-PC7/CD3-AA700/CD45-AA750 и CD5-FITC/CD23-PE/CD19-ECD/CD45-PC5/CD27-PC7. Распределение антител по каналам флуоресценции проводили в соответствии с принципами формирования панелей для многоцветных цитофлуориметрических исследований [12]. Процесс дифференцировки Treg изучался по экспрессии на поверхности Treg рецепторов CD45R0 и CD62L. Исследовался состав следующих субпопуляций Treg: наивные (CD45R0-CD62L+Treg), Treg центральной памяти (CD45R0+CD62L+Treg), эффекторной памяти (CD45R0+CD62L-Treg) и терминально-дифференцированные Treg (CD45R0-CD62L-Treg). Уровни экспрессии поверхностного рецептора СD25 оценивали по средней интенсивности флуоресценции (MFI). Пробоподготовку выполняли по стандартной методике [13]. Анализ окрашенных клеток проводили на проточном цитофлуориметре Navios (Beckman Coulter, USA) Красноярского регионального центра коллективного пользования ФИЦ КНЦ СО РАН. Обработку полученных цитофлуориметрических результатов осуществляли с помощью программ Navios Software v. 1.2 и Kaluzav. 2.1.1 (Beckman Coulter, USA). В каждой пробе анализировали не менее 50 000 лимфоцитов.

## Статистический анализ

Описание полученных данных производили с помощью подсчета медианы (Ме) и интерквартильного размаха в виде 1–3 квартилей (Q1–Q3). Для определения характера распределения полученных данных использовали критерий Шапиро–Уилка. Статистическую значимость различий между исследуемыми показателями больных и контрольной группы оценивали по непараметрическому критерию Манна–Уитни (Mann–Whitney U-test). Статистическую значимость различий в динамике после радионуклидного лечения определяли с помощью критерия Вилкоксона (Wilcoxon matched pairs test). Статистический анализ осуществляли в пакете прикладных программ Statistica 8.0 (StatSoftInc., 2007).

## Этическая экспертиза

Протокол исследования рассмотрен и одобрен локальным этическим комитетом ФГБОУ ВО «Красноярский государственный медицинский университет им. профессора В.Ф. Войно-Ясенецкого» Минздрава России (протокол № 72/2016 от 09.11.2016) и КГБУЗ «Краевая клиническая больница» (протокол № 124 от 07.04.2016).

## РЕЗУЛЬТАТЫ

В исследование включены 36 женщин с рецидивом БГ, средний возраст 46,34±14,32 года, которые наблюдались в эндокринологическом центре КГБУЗ «Краевая клиническая больница» и получали консервативное лечение тиамазолом с дебюта заболевания. При верификации заболевания манифестный гипертиреоз диагностирован у 29 (80,00%) и субклинический гипертиреоз — у 7 (20,00%) пациентов соответственно, ТТГ=0,01 мЕд/л (0,001–0,03), свТ3=7,34 пмоль/л (5,11–12,32), свТ4=32,09 пмоль/л (25,63–38,12) и ТТГ=0,02 мЕд/л (0,004–0,11), свТ3=5,01 пмоль/л (3,23–5,36), свТ4=13,27 пмоль/л (11,25–18,42). На момент манифестации заболевания медиана уровня антител к рТТГ у пациентов с БГ статистически значимо не отличалась в зависимости от степени тяжести гипертиреоза и составила 14,52 мЕд/л (7,95–23,15). Медиана продолжительности консервативного лечения тиамазолом составила 5 мес (2–9), при этом у 27 (75,00%) рецидив гипертиреоза развивался в течение 2 мес, а у 9 (25,00%) — спустя 7–12 мес после достижения медикаментозного эутиреоза.

При планировании радионуклидного лечения все пациентки с рецидивом заболевания принимали поддерживающую дозу тиамазола и находились в состоянии медикаментозного эутиреоза: медианы уровней ТТГ, свТ3 и свТ4 на фоне приема поддерживающей дозы тиреостатика составили соответственно 0,21 мЕд/л (0,11–0,67), 4,21 пмоль/л (3,42–4,91) и 15,29 пмоль/л (11,54–19,81). Уровень антител к рТТГ при купировании рецидива гипертиреоза статистически значимо не отличался от показателей, установленных в дебюте заболевания, и составил 12,91 (5,59–21,32) мЕд/л. Клинико-лабораторная характеристика пациентов с БГ на момент обследования до РЙТ и в динамике после радионуклидного лечения представлена в таблице 1.

**Table:** Таблица 1. Клинико-гормональная характеристика пациентов с болезнью Грейвса в динамике после радионуклидного лечения (РЙТ), Ме, (Q1–Q3)Table 1. Clinical and hormonal characteristics of patients with Graves' disease in dynamics after radionuclide treatment (RIT), Me, (Q1–Q3) Примечание. p — статистически значимые различия с контрольными величинами; p2 — статистически значимые различия с показателями пациентов до РЙТ; p3 — статистически значимые различия с показателями пациентов через 1 мес после РЙТ. свТ4 — свободный тироксин, свТ3 — свободный трийодтиронин, рТТГ — рецептор тиреотропного гормона.* Клинико-гормональные показатели до РЙТ соответствуют обследованию пациентов с БГ через 14 дней после отмены поддерживающей дозы тиамазола.

Показатель	Контроль n=42	Пациенты с БГ в динамике после радионуклидного лечения(n=36)
до РЙТ	через 1 мес	через 3 мес	через 6 мес
1	2	3	4	5
ТТГ, мЕд/л	1,14 (0,86–1,56)	0,07 (0,01–0,45) p1<0,001	7,23 (6,23–10,72) p1=0,010 p2 <0,001	2,36 (0,68−7,81) p1<0,001 p3<0,001	2,12 (0,99−3,44) p1<0,001 p3<0,001
свТ3, пмоль/л	4,14 (2,91–5,52)	5,21 (4,31–6,91) p1<0,001	2,52 (1,68–3,87) p1=0,010 p2 <0,001	3,21 (2,21−4,25) p1=0,010	3,42 (2,63−4,57) p2<0,001
свТ4, пмоль/л	14,12 (12,41–15,83)	16,21 (14,82–18,06) p1<0,001	10,21 (7,16–11,05) p1 <0,001 p2 <0,001	12,31 (9,56–14,61) p1=0,011 p2<0,001	13,22 (12,02−15,78) p2<0,001 p3<0,001
Антитела к рТТГ,МЕ/л	0,21 (0,11-0,35)	12,41 (5,42–21,12) p1<0,001	1,75 (0,49–5,33) p1 <0,001 p2 <0,001	2,61 (1,81–3,43) p1<0,001 p2<0,001	1,42 (0,81–2,56) p1<0,001 p2<0,001
Объем ЩЖ, мл	9,91 (9,41−12,63)	23,42 (17,61–27,81) p1<0,001	16,23 (11,29–18,72) p1 <0,001 p2 <0,001	13,51 (9,21–17,06) p1<0,001 p2<0,001	9,24 (7,66–11,25) p1<0,001 p2<0,001 p3<0,001

На фоне отмены антитиреоидной терапии в течение 2 нед до радионуклидного лечения у большинства обследуемых пациентов развился субклинический гипертиреоз с сохраняющимся высоким уровнем аутоантител к рТТГ, титр которых статистически значимо не изменялся в сравнении со значениями, установленными в состоянии достигнутого медикаментозного эутиреоза. Медиана терапевтической активности 131I при радионуклидном лечении обследованных пациентов с БГ cоставила 500 мБк (400–700). У всех пациентов клинико-лабораторный симптомокомплекс пострадиационного гипотиреоза развивался на 3–4-й неделе после РЙТ, и при обследовании через 1 мес после радионуклидного лечения медиана уровня ТТГ соответствовала субклиническому гипотиреозу, а уровень аутоантител к рТТГ был статистически значимо снижен относительно значений, установленных до радионуклидного лечения, но оставался повышенным относительно контроля. При обследовании через 3 мес после радионуклидного лечения все пациенты находились в состоянии компенсированного пострадиационного гипотиреоза. При контрольном клинико-гормональном обследовании через 6 мес после радионуклидного лечения все пациенты сохраняли компенсированный пострадиационный гипотиреоз. Уровень антител к рТТГ статистически значимо снижался в течение всего пострадиационного периода, но оставался повышенным относительно контрольных значений в исходе радионуклидного лечения. При исследовании содержания Treg (CD3+CD4+CD127LowCD25High) в крови у пациентов с БГ обнаружено, что еще до проведения радионуклидного лечения у больных относительно контрольных значений было снижено абсолютное, но не процентное содержание Treg (табл. 2).

**Table:** Таблица 2. Содержание различных субпопуляций регуляторных Т-лимфоцитов в крови у пациентов с болезнью Грейвса в динамике после радионуклидного лечения (РЙТ), Ме (Q1–Q3)Table 2. The content of various subpopulations of regulatory T-lymphocytes in the blood of patients with Graves' disease in dynamics after radionuclide treatment (RIT), Me (Q1–Q3) Примечание. p1 — статистически значимые различия с контрольными величинами; p2 — статистически значимые различия с показателями пациентов до РЙТ; p3 — статистически значимые различия с показателями пациентов через 1 мес после РЙТ.

Субпопуляции регуляторных Т-лимфоцитов	Содержание регуляторных Т-лимфоцитов в крови в норме (n=42) и при болезни Грейвса в динамике после радионуклидного лечения (n=36)
контроль 1	до РЙТ 2	через 1 мес 3	через 3 мес 4	через 6 мес 5
CD3+CD4+CD127LowCD25High, 109/л	0,069(0,059–0,117)	0,030(0,013–0,067) p1<0,001	0,025(0,013–0,065) p1<0,001	0,022(0,013–0,033) p1<0,001	0,022(0,017–0,035) p1<0,001 р2=0,008
CD3+CD4+CD127LowCD25High, %	1,55(0,83–3,09)	1,33(0,69–3,03)	1,36(0,65–2,56)	0,95(0,79–1,58)	1,00(0,77–2,04) р2=0,015
Treg CD45R0-CD62L+, %	0,36(0,26–0,69)	0,124(0,043–0,223) p1<0,001	0,146(0,067–0,234) p1<0,001	0,001(0,0007–0,002) p1,2,3<0,001	0,001(0,0006–0,016) p1,2,3<0,001
Treg CD45R0+ CD62L+, %	0,60(0,32–0,89)	0,71(0,58–0,99)	0,72(0,40–0,95)	0,50(0,34–0,60)	0,82(0,50–1,08)
Treg CD45R0+ CD62L-, %	0,41(0,21–0,55)	0,47(0,29–0,63)	0,41(0,27–0,53)	0,43(0,25–0,57)	0,15(0,12–0,32) р2=0,018 р3=0,012
Treg CD45R0-CD62L-, %	0,145(0,124–0,207)	0,021(0,009–0,031) p1<0,001	0,030(0,017–0,052) p1<0,001	0,001(0,0006–0,002) p1,2,3<0,001	0,001(0,0007–0,002) p1,2,3<0,001

В течение 6 мес после радионуклидного лечения абсолютное число Treg оставалось сниженным. Более того, к концу периода наблюдения (6-й мес РЙТ) у обследованных пациентов выявлялось понижение абсолютного и процентного содержания Treg относительно исходных значений. Процентный уровень наивных Treg (CD45R0-CD62L+) у пациентов с БГ понижен относительно контрольного диапазона до и в течение всего периода после радионуклидного лечения, но через 3 и 6 мес наблюдается значительное снижение клеток с данным фенотипом как относительно значений, выявляемых у больных в период до, так и через 1 мес после РЙТ. Количество Treg центральной (CD45R0+CD62L+) и эффекторной (CD45R0+CD62L-) памяти в крови у пациентов с БГ соответствовало уровням контрольного диапазона. Однако через 6 мес после РЙТ у обследованных пациентов обнаружено понижение уровня Treg эффекторной памяти (CD45R0+CD62L-) относительно значений, выявляемых в период до и через 1 мес после радионуклидного лечения. Процентное содержание терминально-дифференцированных (CD45R0-CD62L-) Treg у больных снижено относительно контрольного диапазона в течение всего периода наблюдения. В то же время уровень клеток с данным фенотипом дополнительно понижался в период через 3 и 6 мес после РЙТ относительно значений, выявляемых до и через 1 мес после радионуклидного лечения.

При исследовании уровня экспрессии CD25 (MFI) на мембране различных субпопуляций Treg в крови у пациентов с БГ были установлены значительные отличия от контрольных значений, которые зависят от сроков наблюдения в пострадиационном периоде (табл. 3).

**Table:** Таблица 3. Уровень экспрессии CD25 (MFI) на мембране различных субпопуляций регуляторных Т-лимфоцитов (в относительных единицах) в крови у пациентов с болезнью Грейвса в динамике после радионуклидного лечения (РЙТ), Ме (Q1–Q3)Table 3. The level of expression of CD25 (MFI) on the membrane of various subpopulations of regulatory T-lymphocytes (in relative units) in the blood of patients with Graves' disease in dynamics after radionuclide treatment (RIT), Me (Q1–Q3) Примечание. p1 — статистически значимые различия с контрольными величинами; p2 — статистически значимые различия с показателями пациентов до РЙТ; p3 — статистически значимые различия с показателями пациентов через 1 мес после РЙТ.

Субпопуляции регуляторных Т-лимфоцитов	Уровень экспрессии CD25 (MFI) в норме (n=42) и при болезни Грейвса в динамике после радионуклидного лечения (n=36)
контроль 1	до РЙТ 2	через 1 мес 3	через 3 мес 4	через 6 мес 5
Treg CD45R0-CD62L+	5,11 (3,46–8,35)	0,01 (0,007–3,24) p1<0,001	0,01 (0,006–0,02) p1<0,001	0,01 (0,007–0,02) p1<0,001	0,01 (0,005–0,02) p1<0,001
Treg CD45R0+CD62L+	5,91 (5,09–7,48)	5,23 (4,67–8,62)	5,71 (5,21–6,02)	5,22 (4,95–5,39) p1=0,034	5,15 (0,05-5,77) p1=0,019
Treg CD45R0+CD62L-	5,81 (4,68–7,29)	5,15 (1,77–6,44)	4,97 (4,64–6,02)	5,37 (2,32–5,84)	5,01 (0,93–6,78)
Treg CD45R0-CD62L-	4,87 (4,13–6,22)	0,01 (0,005–0,02) p1<0,001	0,01 (0,004–0,02) p1<0,001	0,01 (0,006–0,02) p1<0,001	0,01 (0,006–0,02) p1<0,001

До радионуклидного лечения в периферической крови пациентов с БГ снижен уровень экспрессии MFI рецептора CD25 относительно контрольных значений на наивных (CD45R0-CD62L+) и терминально-дифференцированных (CD45R0-CD62L-) Treg, остальные субпопуляции Treg экспрессировали рецептор CD25 в соответствии с показателями контрольной группы. Через 1 мес после радионуклидного лечения, несмотря на развитие пострадиационного гипотиреоза, у обследуемых пациентов зафиксированы аналогичные статистически значимые изменения в экспрессии CD25 на отдельных субпопуляциях Treg. Однако наиболее значимые изменения в уровнях экспрессии рецептора CD25 на поверхности отдельных субпопуляций Treg у пациентов с БГ были обнаружены через 3 и 6 мес после радионуклидного лечения. Так, через 3 и 6 мес после приема терапевтической активности 131I, на фоне компенсированного пострадиационного гипотиреоза, в крови пациентов с БГ остаются пониженными уровни MFI CD25 на поверхности наивных и терминально-дифференцированных Treg, но добавляется понижение экспрессии данного маркера на мембране Treg центральной памяти (CD45R0+CD62L+). Уровень MFI CD25 на поверхности Treg эффекторной памяти (CD45R0+CD62L-) у пациентов с БГ соответствовал значениям контрольной группы в течение всего периода наблюдения.

## ОБСУЖДЕНИЕ

## Репрезентативность выборок

Набор участников исследования проводился только в эндокринологическом центре КГБУЗ «Краевая клиническая больница». Радионуклидное лечение осуществлялось всем пациентам на базе отделения радионуклидной терапии ФМБА России г. Красноярска. На внешнюю валидность результатов данного исследования могли повлиятьиндивидуальные иммуногенетические аспекты, связанные со степенью дефекта Treg у пациентов с БГ, а также эпигенетические факторы в каждом конкретном случае заболевания.

## Сопоставление с другими публикациями

Treg являются субпопуляцией T-клеток, которая обладает супрессорной активностью и занимает центральное место в поддержании иммунологической толерантности и ограничении иммунного ответа [14]. В предыдущих исследованиях было подтверждено наличие выраженного дефекта Treg при БГ, который заключается в снижении количества Treg и повышении соотношения Th/Treg как до, так и после лечения [15]. Отмечено, что у пациентов с БГ, получающих консервативное лечение тиамазолом в состоянии стойкого медикаментозного эутиреоза более 12 мес, увеличение количества Treg в периферической крови не приводит к снижению доли активированных Th-клеток [16]. Абсолютное снижение Treg в ходе развития БГ и тенденция к восстановлению их уровня после лечения антитиреоидными препаратами у пациентов с БГ были установлены и ранее. Авторами выявлена корреляция низкого уровня Treg с некоторыми клиническими проявлениями тиреотоксикоза, в частности, такими, как экзофтальм и/или снижение веса в дебюте гипертиреоза Грейвса [17]. В других публикациях обсуждается, что радионуклидное лечение оказывает специфическое иммунное воздействие. В 30-дневном проспективном исследовании с участием 27 пациентов с болезнью Грейвса, из которых 11 получали антитиреоидные препараты, было показано, что разница в соотношении числа Treg и инвариантных Т-клеток естественных киллеров после РЙТ в группе больных была выше, чем в группе контроля. Авторы также отмечают и снижение супрессорной функции Treg у обследуемых больных с БГ после РЙТ, объясняя выявленные изменения усилением иммунной активности [18]. Клинические исследования содержания отдельных субпопуляций Treg после радионуклидного лечения отсутствуют. Но к настоящему времени накоплены убедительные данные о том, что положительный титр антител к рТТГ у пациентов с болезнью Грейвса может сохраняться длительное время после тиреоидэктомии и радионуклидного лечения [19], что согласуется и с полученными нами данными. Настоящее исследование демонстрирует, что радионуклидное лечение оказывает иммуносупрессивное воздействие на систему адаптивного иммунитета, что проявляется в пониженном содержании Treg и низком уровне экспрессии рецептора CD25 на мембране отдельных субпопуляций Treg. Исходя из результатов исследования установлено, что у пациентов с БГ в периферической крови в динамике после радионуклидного лечения снижено количество наивных и терминально-дифференцированных Treg. После проведения РЙТ у пациентов с БГ количество данных субпопуляций Treg не восстанавливается и достигает минимальных значений к 6-му месяцу после радионуклидного лечения. Пониженное содержание Treg у пациентов с БГ было выявлено и до радионуклидного лечения, независимо от титра антител к рТТГ, функции ЩЖ и предшествующей консервативной терапии тиамазолом. Причем низкий уровень Treg у пациентов с БГ до РЙТ определяется несмотря на прогрессивное снижение титра антител к рТТГ, которое наблюдалось с 1-го месяца после терапии 131I, что свидетельствует о сохранении количественного дефекта Treg, независимо от терапевтического воздействия 131I и устранения антигенного стимула. Низкое абсолютное число Treg у пациентов с БГ сразу, через 1 и 3 мес после РЙТ и еще большее их снижение через 6 мес поcле радионуклидного лечения является аргументом в пользу иммуносупрессивного воздействия 131I. В то же время обращает на себя внимание, что у пациентов с БГ общее процентное число Treg (CD3+CD4+CD127LowCD25High) и количество Treg центральной и эффекторной памяти сохраняется в диапазоне нормальных значений в течение длительного времени, что может объясняться большей резистентностью данных субпопуляций Treg к воздействию 131I в сравнении с остальными исследуемыми субпопуляциями Treg. Следует отметить и то, что Treg центральной памяти характеризуются как самая долгоживущая субпопуляция Т-лимфоцитов с длительным периодом циркуляции по сравнению с другими клетками памяти [14]. В свою очередь, Treg эффекторной памяти не способны мигрировать в периферические лимфоидные органы за счет отсутствия экспрессии CD62L, но проявляют более выраженную функциональную активность. Таким образом, длительное сохранение Treg памяти обоих типов у пациентов с БГ после радионуклидного лечения может объясняться их эффекторными свойствами и степенью дифференцировки.

Обнаружено, что в период до проведения РЙТ у пациентов с БГ снижен уровень экспрессии MFI СD25 именно на наивных и терминально-дифференцированных Treg. После РЙТ уровень экспрессии CD25 на наивных и терминально-дифференцированных Treg не восстанавливается, и, более того, на 3-м и 6-м месяцах после радионуклидного лечения выявляется снижение экспрессии данного маркера на мембране Treg центральной памяти. Известно, что результатом связывания CD25 является активация STAT (signal transducer and activator of transcription), mTOR (mammalian target of rapamycin) и различных MAPK (mitogen-activated protein kinase, митоген-активируемая протеинкиназа), что проявляется в усилении пролиферативной активности и выживаемости клеток [20]. Следовательно, снижение количества наивных и терминально-дифференцированных Treg у пациентов с БГ может определяться низким уровнем экспрессии рецептора CD25 и, соответственно, пониженным уровнем пролиферативного ответа, индуцируемым IL-2. Выявленные изменения согласуются с предположением некоторых авторов о том, что при БГ существуют некоторые генетические изменения в гене CD25, которые предрасполагают к развитию аутоиммунного процесса путем нарушения функции Treg и адекватного развития периферической толерантности [21].

## Клиническая значимость результатов

Выявленные закономерности в исходном дефекте Treg, его реализации на фоне двухнедельной отмены тиамазола перед РЙТ и срыва иммунологической толерантности с развитием субклинического гипертиреоза, а также иммуносупрессивное воздействие радионуклидного лечения на содержание и уровень экспрессии CD25 на мембране различных субпопуляций Treg свидетельствуют о стойком дефекте Treg при БГ и позволяют выделить субпопуляции наивных (CD45R0-CD62L+) и терминально-дифференцированных (CD45R0-CD62L-) Treg как потенциальные мишени для разработки иммунотропной терапии заболевания.

## Ограничения исследования

Выраженность дефекта Treg также зависит от уязвимости иммунорегуляторных генов и их конечных продуктов транскрипции, которые расположены на поверхности Treg, таких как FOXP3, CD25 и CTLA-4. Эти гены и их продукты участвуют в обеспечении надлежащего поддержания периферической толерантности (FOXP3 и CD25) и установлении соответствующей ко-стимуляции (CTLA-4) [2]. В связи с этим клинически проявление дефекта Treg при БГ может различаться индивидуально, в зависимости от накопленных эпигенетических факторов, у каждого конкретного пациента.

## Направления дальнейших исследований

Необходимы дальнейшие иммуногенетические исследования для уточнения ассоциации степени дефекта Treg с аллелями генов системы HLA II класса и экспрессией HLA-DR на клетках иммунной системы, что поможет идентифицировать пациентов с БГ с более выраженным дефектом иммунорегуляции в клинической практике.

## ЗАКЛЮЧЕНИЕ

При анализе содержания субпопуляций Treg у пациентов с БГ установлено понижение уровня наивных Treg (по-видимому, за счет нарушения процессов дифференцировки в тимусе) и терминально-дифференцированных Treg (за счет процессов созревания и выживаемости), которые дополняются пониженным уровнем экспрессии MFI рецептора CD25 на поверхности этих клеток и не зависят от компенсации гипертиреоза, титра антител к рТТГ, предшествующей консервативной терапии тиамазолом и радионуклидного лечения. Расширение знаний о роли cубпопуляций Treg и значении наивных (CD45R0-CD62L+) и терминально-дифференцированных (CD45R0-CD62L-) Treg в иммунопатогенезе БГ позволяет не только лучше понять механизмы рецидива и прогрессирования аутоиммунного процесса, но и помогает наметить дальнейшие пути разработки подходов для иммунотропной терапии.

## ДОПОЛНИТЕЛЬНАЯ ИНФОРМАЦИЯ

Согласие пациентов. Все исследования выполнены с информированного согласия испытуемых и в соответствии с Хельсинкской декларацией Всемирной ассоциации «Этические принципы проведения научных медицинских исследований с участием человека» с поправками 2013 г. и «Правилами клинической практики в Российской Федерации», утвержденными Приказом Минздрава РФ от 19.06.2003 г. № 266.

Источники финансирования. Исследование выполнялось на базе лаборатории молекулярно-клеточной физиологии и патологии Федерального исследовательского центра «Красноярский научный центр Сибирского отделения Российской академии наук», обособленное подразделение «НИИ медицинских проблем Севера».

Конфликт интересов. Авторы декларируют отсутствие явных и потенциальных конфликтов интересов, связанных с содержанием настоящей статьи.

Участие авторов. Дудина М.А. — получение, анализ данных, интерпретация результатов, написание статьи; Савченко А.А. — концепция и дизайн исследования, внесение в рукопись существенной (важной) правки с целью повышения научной ценности статьи; Догадин С.А. — концепция и дизайн исследования, внесение в рукопись существенной (важной) правки с целью повышения научной ценности статьи; Борисов А.Г. — интерпретация результатов, внесение в рукопись существенной (важной) правки с целью повышения научной ценности статьи; Беленюк В.Д. — получение и интерпретация результатов. Все авторы одобрили финальную версию статьи перед публикацией, выразили согласие нести ответственность за все аспекты работы, подразумевающую надлежащее изучение и решение вопросов, связанных с точностью или добросовестностью любой части работы.
